# National Eye and Ear Infirmary, Dublin

**Published:** 1889-05-18

**Authors:** 


### National Eye and Ear Infirmary, Dublin.

THE annual report of the National Eye and Ear Infirmary, Molesworth-street, Dublin, has just been published, and in it is given some interesting details of the history of the charity. It appears that the institution is the oldest of the kind in Ireland, having been founded in the year 1814. It is interesting to note that about this time several similar charities were founded in the United Kingdom. The Royal London Ophthalmic Hospital, founded in 1804, is the oldest, and after that comes the West of England Eye Infirmary (Exeter), founded in 1808, the Bristol Eye Hospital, founded in 1810, the Bath Eye Infirmary, founded in 1811, and the Manchester Royal Eye Hospital, which was founded in the same year as the first Irish Eye Hospital. There are twenty-nine other ophthalmic institutions in Great Britain, all of which are of later date. The career of the National Eye and Ear Infirmary in Dublin has been a chequered one. It owes its origin to the indefatigable energy of Mr. Ryll, a surgeon in His Majesty's navy, and it received the patronage of the Earl of Whitworth, then Lord-Lieutenant; and Mr. Peel (afterwards Sir Robert) became president of the institution. About the year 1827 Surgeon Ryll was suddenly called away to England, and on his departure the institution almost collapsed, and the house in which it had been established had to be given up, but Surgeon Morrison, who had devoted much time and attention to ophthalmic surgery, came to the rescue at this juncture, and a small house in Gloucester-street was rented. A short period of prosperity now ensued, and a couple of years afterwards a house was rented in Cuffe-street, at that time a fashionable locality. The charity flourished here, doing much good, until the unfortunate year 1848, when, doubtless from the disturbed state of the country, funds fell off, and the charity languished, although it had not altogether closed its doors. For the next twenty years the charity was anything but prosperous, but the devotion of its medical staff and a few others still retained a spark of vitality in the work, and in the year 1872 it was considered advisable to try and extend its sphere of usefulness. A special appeal, to which a generous response was made, enabled the governors to transfer the infirmary from the small house in Cuffe-street to 97, St. Stephen's Green. The institution then grew rapidly, so that in five or six years much larger premises and a completely appointed hospital became a necessity. The charity once more made a move, and in 1877 the committee of management purchased the interest in the lease of the premises in Molesworth-street now occupied by the hospital. Since the foundation of the charity the Lord-Lieutenant for the time being has always filled the office of patron. Last year 377 patients were admitted into the wards of the hospital, and 2,203 were treated in the out-patient department. The receipts were £921 10s. 4d., and the expenditure £966 9s. 11½d., which gives an average cost of £31 14s. 4d. for each bed occupied after deducting 1s. for each out-patient. The Dublin Hospital Sunday Fund liberally supports the charity and the Corporation of the city of Dublin makes an annual grant of £100 to its funds.

